# Furnace Heat

**Published:** 1874-11

**Authors:** 


### FURNACE HEAT.

Much has been written about the injurious effects of the dry, oppressive heat thrown out by the ordinary furnace—injurious to the woodwork of the house, injurious to the furniture, and ruinous to the health of all, except to the most robust. Much of this ill-result is from the smallness of the surface of the fire-pot, making it necessary to keep the furnace red hot all the time, in order to give out enough heat. If this fire-pot was made to present a surface equal to six, eight, or ten square feet, or more, the furnace need never be red hot, and thus the objection to the air being "burnt up" by coming in contact with red hot iron before it is used for heating purposes, would be almost entirely removed. With such broad-surfaces fire-pots, chestnut-sized coal could be used, and even smaller, as in river steamboats, costing a dollar or two less a ton.

A very common mistake is made, and much fuel wasted, in the manner of replenishing coal fires both in furnaces and grates. They should be fed with a little coal at a time, and often; but servants, to save time and trouble, put on a great deal at once, the first result being that almost all the heat is absorbed by the newly put-on coal, which does not give out heat until it has itself become red hot. Hence, for a while, the room is too cold; but when it becomes fairly aglow, the heat is insufferable.

The time to replenish a coal fire is as soon as the coals begin to show ashes on their surface; then put on nearly enough to show a layer of black coal covering the red. This will soon kindle, and as there is not much of it, an excess of heat will not be given out.

Many almost put out the fire by filling the grate as soon as fresh coal is put on, thus leaving all the heat in the ashes, when it should be sent to the new supply of coals. The time to "stir the fire" is just when the newly laid-on coal is pretty well kindled. This method of managing a coal fire is "troublesome;" but it saves fuel gives a more uniform heat, and prevents the discomfort of alternations of heat and cold above referred to. The method employed in the construction of the furnace is of the greatest importance. The flues must be very large, or they will not carry off the gases of combustion, and will speedily become stopped up with dust and ashes. The radiating surface must be of enormous size, or the furnace will be inefficient, unless heated red hot. If thus heated, the air will be burnt up, its vitality consumed, and it will be unfit to breathe.

Mr. Alex. M. Lesley, President of the Trades Savings Bank, manufactures a furnace which pleases us. It seems as if it must be harmless, and it certainly is a powerful heater. It is made for coal or wood, and the admirable fuel, "Carbonite," works perfectly in it. No deleterious gases escape to poison the atmosphere. It costs from one hundred to two hundred dollars, and proves to be a very economical heating device. It is called the Gothic, and is very largely used in private dwellings and in public edifices. Its value rests chiefly in the great size of the fire-pot and its immense radiating surface. Both these features are features of economy, and both are features of healthfulness, as we have explained.

Another advantage possessed by this furnace is its simplicity. Our servant girls are not usually practical machinists, and can scarcely be trusted to manage complicated machines. Whenever Bridget is depended upon to attend the furnace, it should be so simple that "the way-faring man, though a fool, need not err therein." Therefore, we can commend Mr. Lesley's furnace. The depth of ignorance must be profound and unfathomable which could fail to keep the "Gothic" in good running order all winter, without accident and without annoyance.

Mr. Lesley's factory is at 224 and 226 West 23d Street, New York, and he will cheerfully mail a description of his furnace to all inquirers. His descriptive circular contains a large number of testimonials from users of the "Gothic" all over the country, and there are few towns and cities in the North where they may not be seen in operation every winter.

